# Public health genomics capacity assessment: readiness for large-scale pathogen genomic surveillance in Canada’s public health laboratories

**DOI:** 10.1186/s12889-022-14210-9

**Published:** 2022-09-24

**Authors:** C. Nadon, M. Croxen, N. Knox, J. Tanner, A. Zetner, C. Yoshida, G. Van Domselaar

**Affiliations:** 1 Division of Enteric Diseases, National Microbiology Laboratory, Public Health Agency of Canada, Winnipeg, Canada; 2 Department of Medical Microbiology and Infectious Diseases, Rady Faculty of Health Sciences, Max Rady College of Medicine, University of Manitoba, Winnipeg, Canada; 3 Alberta Precision Laboratories, Public Health Laboratory, Edmonton, Canada; 4 Department of Laboratory Medicine, University of Alberta, Edmonton, Canada; 5 Li Ka Shing Institute of Virology, University of Alberta, Edmonton, Canada; 6 Division of Science and Technology Cores and Services, Bioinformatics Section, National Microbiology Laboratory, Public Health Agency of Canada, Winnipeg, Canada; 7 Canadian Public Health Laboratory Network COVID Genomics Program, National Microbiology Laboratory, Public Health Agency of Canada, Winnipeg, Canada

**Keywords:** Genomics, Capacity building, Surveillance, SARS-CoV-2, Public health laboratory

## Abstract

**Background:**

Along with rapid diagnostic testing, contact tracing, and public health measures, an effective pandemic response incorporates genomics-based surveillance. Large-scale SARS-CoV-2 genome sequencing is a crucial component of the global response to COVID-19. Characterizing the state of genomics readiness among Canada’s public health laboratories was necessary to inform strategic planning and deployment of capacity-building resources in the early stages of the pandemic.

**Methods:**

We used a qualitative study design and focus group discussions, encompassing both technical and leadership perspectives, to perform an in-depth evaluation of the state of pathogen genomics readiness in Canada.

**Results:**

We found substantial diversity in the state of readiness for SARS-CoV-2 genomic surveillance across Canada. Despite this variability, we identified common barriers and needs in the areas of specimen access, data flow and sharing, computing infrastructure, and access to highly qualified bioinformatics personnel.

**Conclusions:**

These findings enable the strategic prioritization and deployment of resources to increase Canada’s ability to perform effective public health genomic surveillance for COVID-19 and prepare for future emerging infectious diseases. They also provide a unique qualitative research model for use in capacity building.

**Supplementary Information:**

The online version contains supplementary material available at 10.1186/s12889-022-14210-9.

## Background

The global COVID-19 pandemic has created many challenges for Canada’s healthcare and public health systems. Included among these challenges is the implementation of genomic surveillance for SARS-CoV-2. Initially, the sequencing and sharing of an early SARS-CoV-2 virus genome provided the world with critical information about the relatedness of this new virus to previously sequenced coronaviruses and enabled the rapid design of diagnostic targets that are routine in detecting the virus [1]. Genomics provided key evidence to demonstrate that sustained community transmission had occurred in the United States prior to the first detection of cases [2], as well as insights into the initial introduction and local transmission of the virus. [3, 4]. Since then, the emergence and spread of new SARS-CoV-2 variants have illuminated the critical role of genomics in the pandemic response. The ability to track mutations in the SARS-CoV-2 genome and determine their effects on pathogenesis, transmission, and diagnostic and vaccine effectiveness have become high priorities for public health authorities in Canada and worldwide [5–8].

Despite the importance of genomics in informing public health activities during the SARS-CoV-2 pandemic, few countries were prepared to rapidly implement large-scale SARS-CoV-2 genomic surveillance [9]. One notable exception is the successful mobilization of large-scale genomic sequencing in the United Kingdom (via the COVID-19 Genomics UK Consortium (COG-UK)), which features a large decentralized network of public health, academic, and research facilities that were largely well-equipped in advance [10]. In the United States, where pre-existing expertise and equipment were extensive, there were claims that the initially slow genomics rollout was due to systemic problems in sharing samples and data [5]. The World Health Organization’s advice and guidance specify that continuous genomic surveillance is critical for the COVID-19 response for monitoring variants and recommends the shift from virus detection only to genomic-based surveillance for influenza-like illness, acute respiratory infection, and severe acute respiratory infection [8].

**Fig. 1:**
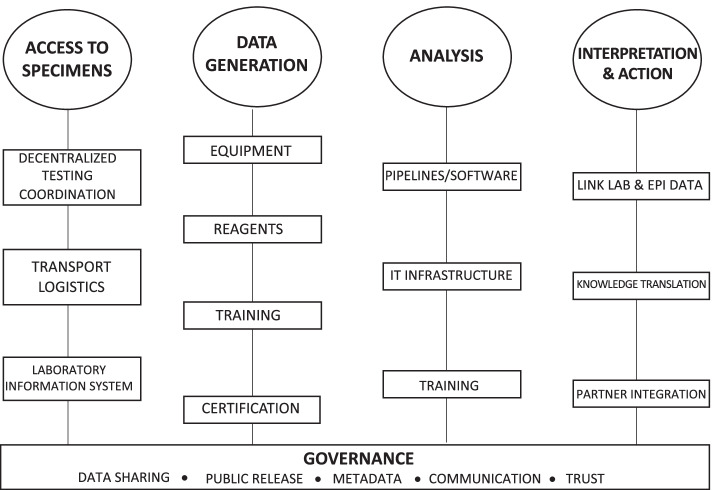
Essential components of genomics capacity building

Prior to the pandemic, pathogen genomics was an active area of research and development in Canada. It had been successfully applied, for example, to investigate tuberculosis transmission in remote settings [11], to characterize carbapenemase-producing Enterobacteriaceae and invasive group A Streptococci in hospitals [12, 13], to support Canada’s largest beef recall due to *E. coli* O157:H7 [14], and to characterize outbreaks of *Salmonella* Heidelberg [15]. Canada had only implemented a national rollout of routine, real-time genomic surveillance for bacterial foodborne diseases. Capacity building for foodborne disease genomic surveillance in Canada occurred over a five-year period (2013–2017). It comprised all four elements of genomics capacity building—data generation, data analysis, results interpretation, and governance—and specifically included activities such as the provision of sequencing instruments for provincial public health laboratories, training for laboratories to generate and analyze the data, and knowledge translation for epidemiologists to interpret and apply genomics results to inform public health decision-making (Fig. 1).

While many early achievements in the implementation of routine genomic surveillance around the world were also in the area of foodborne disease [16–19], the responses to the Ebola and Zika epidemics during 2013–2015 shaped the model for modern genomic epidemiology for emerging diseases in a global setting [20]. Underpinning the initial capacity building for pathogen genomics for public health in Canada was the development of the Integrated Rapid Infectious Disease Analysis platform for genomic epidemiology (IRIDA), which brought bioinformatics infrastructure and pipelines to support genomic activities at the Public Health Agency of Canada’s National Microbiology Laboratory (PHAC-NML) and provincial public health laboratories [21]. As a result of these capacity-building efforts, PHAC-NML and several provincial public health laboratories developed significant expertise in genomics and bioinformatics before the pandemic, and some capacity was in place for genomic surveillance. These previous efforts, including the successful national rollout of routine genomic surveillance for foodborne diseases, meant that Canada was not starting from scratch in implementing pathogen genomics to support the COVID-19 public health response.

In April 2020, Genome Canada launched the Canadian COVID Genomics Network (CanCOGeN) to coordinate a pan-Canadian network for large-scale sequencing of SARS-CoV-2 genomes and host genomes. The Canadian Public Health Laboratory Network (CPHLN) typically coordinates the rollout of laboratory testing for public health purposes in Canada. The CPHLN, comprised of PHAC-NML and the public health laboratories of all ten provinces and three territories (or the laboratories that fill the public health role), has a mandate to assure integrated public health laboratory response to infectious diseases [22]. With the increased scale and complexity of the COVID-19 genomics response, a broader strategy was necessary: CanCOGeN includes in its network the CPHLN plus healthcare partners, academia, industry, research institutes, and sequencing centres [23].

Implementing genomics for the surveillance of infectious diseases is a challenging endeavour, even without the pressures of a pandemic. Genome sequencing is more complex than simply replacing one laboratory test with another. Its use necessitates adequate IT infrastructure, specialized equipment and reagents, the ability to analyze data using bioinformatics, and technical knowledge to interpret it and apply the results in a public health context, along with sustainable funding to maintain capacity over time (Fig. 1). The World Health Organization estimates that countries must overcome at least 22 challenges and barriers for successful genomics implementation, categorized into four groups: organizational, cultural, technical, and scientific [24]. Adding to the complexity is that some barriers are challenging in more than one way; for example, genomic data sharing brings technical challenges as well as cultural and organizational challenges. The degree to which laboratories experience these barriers can vary widely; thus, determining the specific needs and challenges encountered in these four areas is an effective strategy to help inform and prioritize the allocation of resources to maximize genomics capacity.

The Canadian public health system did not foresee the massive scale at which genomics capacity would be needed in Canada’s public health laboratories for the COVID-19 response. The purpose of this study was to assess the state of readiness of Canada’s public health laboratories to perform large-scale genomic surveillance of SARS-CoV-2 in the early stages of the pandemic and to identify specific challenges and opportunities to inform strategic deployment of the capacity necessary to operationalize this crucial public health function at a massive scale. The methods used in this study may also serve as a model for strategic planning for genomics implementation and capacity building by other countries or laboratory networks that wish to build capacity for genomic surveillance of SARS-CoV-2.

## Methods

### Study design

A qualitative study survey was designed and included a survey instrument implemented via focus group discussion to capture each public health laboratory’s priorities and operational landscape, current and planned SARS-CoV-2-genomics-related activities, genomics-based surveillance plans in general (i.e., for other pathogens), and the status of all critical dependencies. Questions were primarily open-ended and intended to generate narrative responses. Questions targeted laboratory leaders/directors, technical personnel, or both.

### Data collection

We invited public health laboratories (or the institutions serving a public health lab function) in all provinces and territories to participate. Participation was by invitation only; there were no exclusion criteria. We conducted our focus group discussions wherein the survey was administered to leadership and technical personnel (as separate groups) for each jurisdiction via video conference interviews or completed in writing. Interviewers recorded responses from the participants to each question. In total, 44 laboratory leaders and technical personnel from 10 laboratories participated in the study.

### Qualitative analysis

The responses to each question were converted into key messages and stratified by jurisdiction (province or territory) and by personnel type (leadership vs. technical) in Excel (Microsoft, USA) following general grounded theory and focus group methodology [25–27]. During the initial review of the data, we coded responses into categories. We then re-examined the responses to identify concepts among the categories, which we assessed for trends, themes, and deviations. In some cases, we tabulated coded responses to generate quantitative analyses and calculated the response proportions as percentages. Not every participant answered every question; these were coded as missing values and were excluded from the analysis. The key messages were communicated back to each interview participant for validation and corrected when needed. Excerpts included herein as quotes or featured as stories were edited for clarity and to de-identify the respondent. Results were visualized in R [28] and RStudio [29] using the tidyverse [30], hrbrthemes [31], treemapify, and ggfittext [32] packages.

## Results

**Fig. 2:**
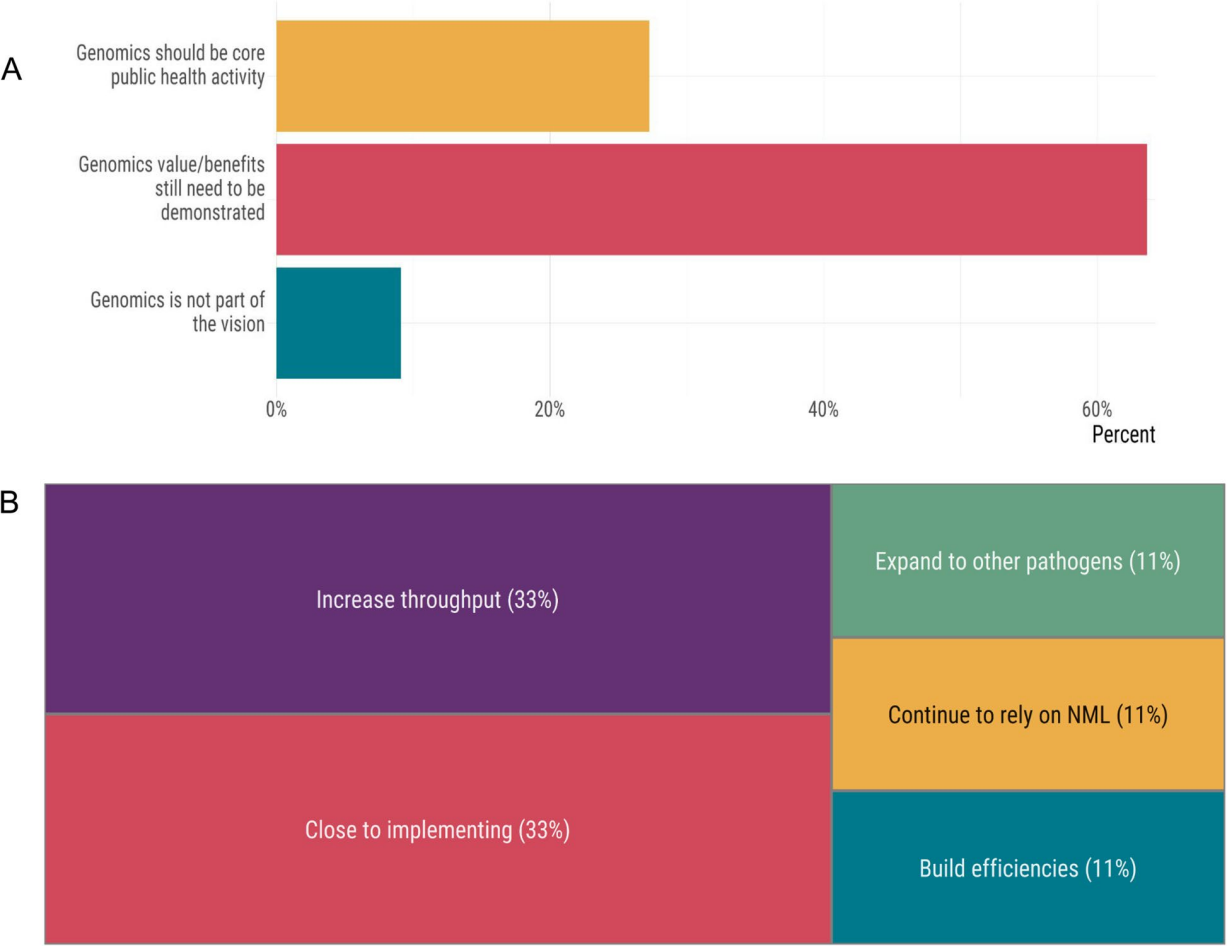
Perception (**A**) and plans (**B**) for genomics for SARS-Cov-2 genomics in Canadian public health laboratories

Although initial capacity was generally low relative to the scale required (with some exceptions), genomics for SARS-CoV-2 was considered a priority by most public health laboratories, even before the emergence of variants of concern. The perceived role of genomics in the national COVID-19 response was evaluated as an indication of any cultural barriers to genomics implementation. In the early phase of the pandemic (i.e., before the emergence of variants of concern), SARS-CoV-2 genomics was considered a priority by 70% of public health laboratories (see supplemental data). More than two-thirds of the labs either did not consider SARS-CoV-2 genomics as part of their vision or believed that the value or that genomics had yet to demonstrate its value. In contrast, fewer than one-third reported that genomics should be a core part of public health laboratory activities (Fig. 2a). This viewpoint likely reflects the perception of genomic surveillance overall and initial capacity-building efforts in the years prior to the pandemic. Since this study took place before the emergence of variants of concern and the availability of vaccines (and thus the need to monitor variants for vaccine escape), it is perhaps not surprising that laboratories viewed genomics as a lower priority compared to diagnostic testing, which was required at a scale never before encountered in Canada, and many respondents noted it was consuming most of their resources and attention during the early phase of the pandemic. Genomics was considered a complementary strategy to support public health surveillance, with primary applications in outbreak investigations and tracking important mutations impacting, for example, virulence or the performance of diagnostic tests. Overall, given the scale of the pandemic, capacity across all public health labs was low, with 44% of the labs reporting having little or no capacity and 56% reporting having at least some or moderate capacity. Several provinces had operational genomic surveillance programs or were in the process of transforming their genomics research programs into routine operations. However, reliance on external sequencing centres was required and, for some labs, remains essential. Some laboratories could perform genome sequencing in-house, while others relied on a sequencing partner to carry out SARS-CoV-2 sequencing on their behalf. At the time we administered the survey, PHAC- NML performed SARS-CoV-2 sequencing for six of the 13 jurisdictions in Canada. One-third of all labs reported being very close to implementing SARS-CoV-2 sequencing; another third had implemented genomics and were focused on increasing current genomics throughput. One lab planned to rely on PHAC-NML to perform sequencing on their behalf indefinitely (Fig. 2b).

**Fig. 3:**
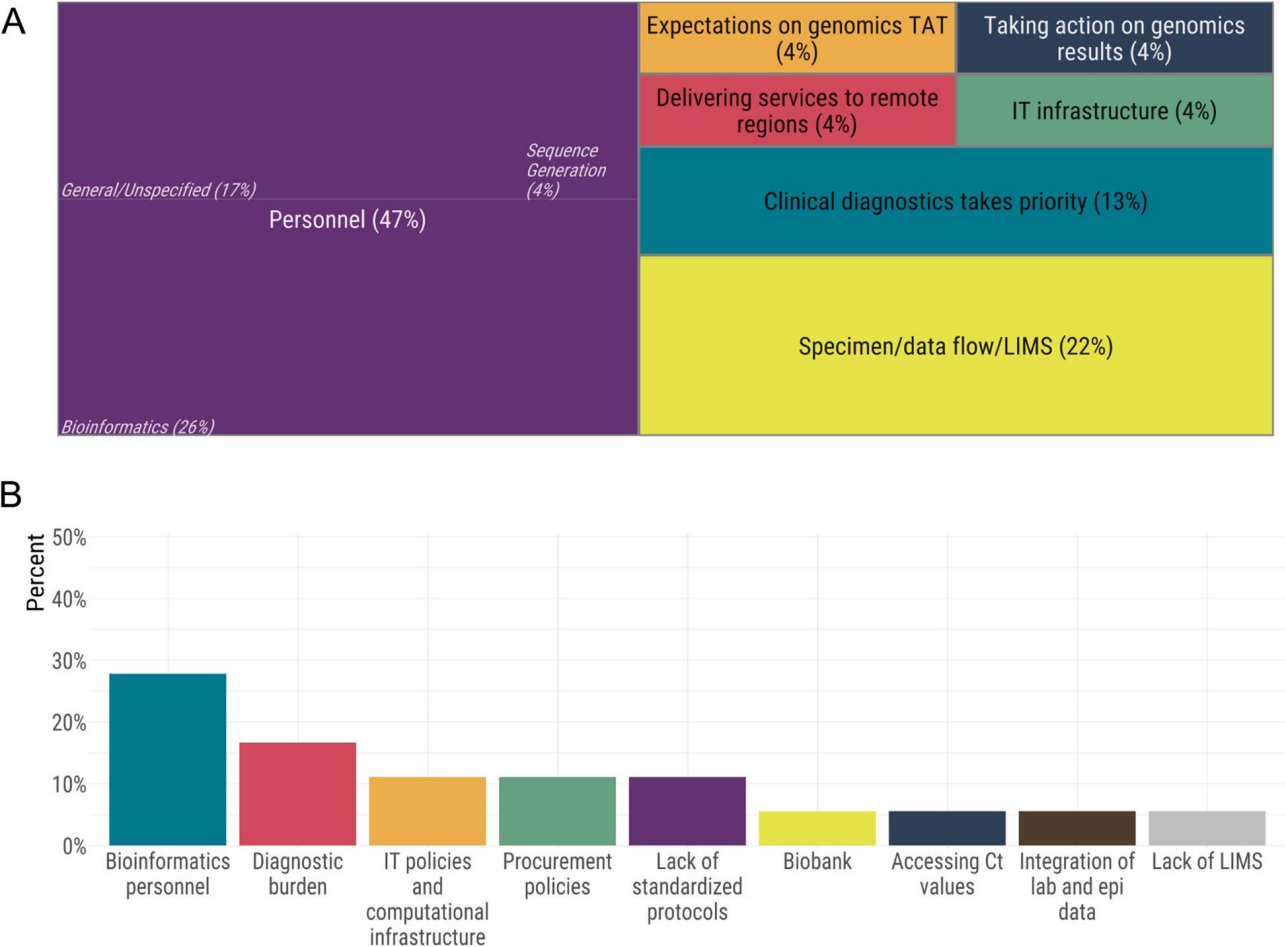
Barriers to (**A**) and pressures of (**B**) implementing genomics experienced by public health laboratories

**Fig. 4:**
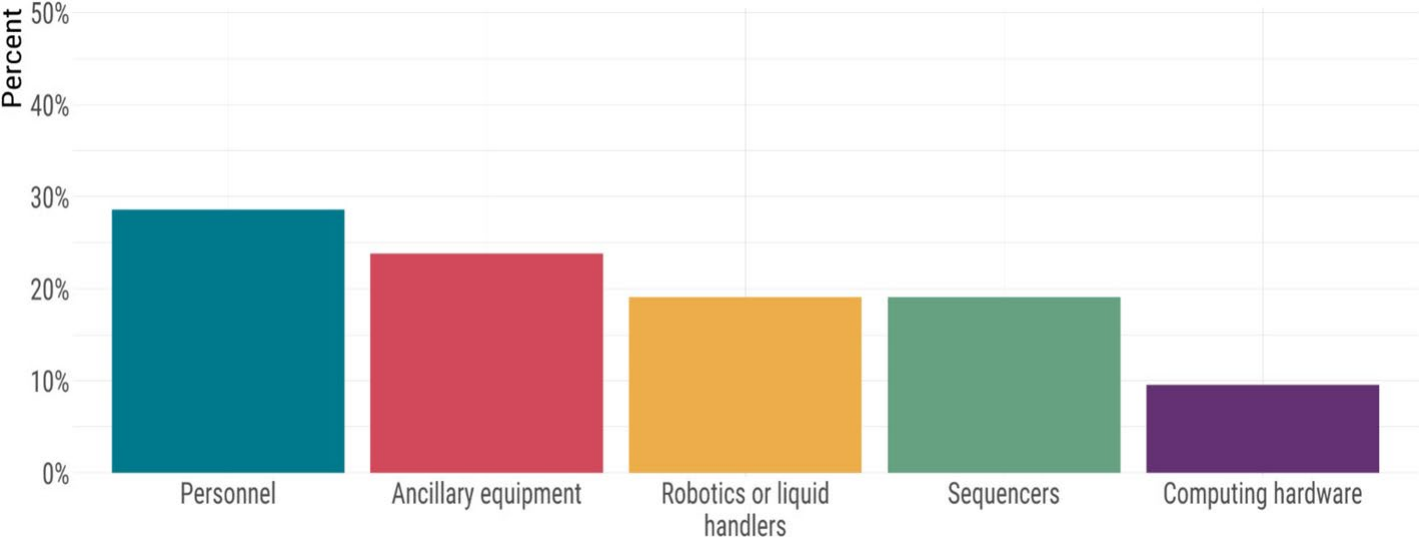
The needs of public health laboratories in order to increase sequencing throughput

The biggest challenge in implementing genomics for SARS-CoV-2, or increasing existing genomics throughput, was the lack of highly qualified personnel, and logistical and computing challenges were also prominent. Overall, several themes emerged among the major challenges faced by public health laboratories (Fig. 3). By far, the challenge most frequently reported by laboratory leaders was a lack of personnel (48%) (Fig. 3a). The participants specified the majority of their personnel needs were for highly qualified personnel in the field of bioinformatics. In many jurisdictions, the provision of diagnostic testing, sample collection, and epidemiological and clinical data collection is part of a very complex and sometimes fragmented landscape; accordingly, the participants reported a lack of a Laboratory Information Management Systems (LIMS) as a commonly encountered challenge (22%). The laboratory leaders also reported that the scale of the pandemic placed diagnostic testing in high demand, consuming much existing laboratory capacity. This presented another major challenge in the implementation of genomic surveillance. Regarding technical expertise, lack of bioinformatics capacity and personnel was also the biggest pressure they faced, followed by the extremely high pressure of delivering diagnostic tests at a massive scale. Technical personnel also reported encountering additional roadblocks, such as the lack of standardized protocols and cumbersome or restrictive IT and/or procurement policies (Fig. 3b). While several labs had implemented some sequencing for SARS-CoV-2, the throughput was insufficient to support routine genomic surveillance. Access to highly qualified personnel was the most frequently cited barrier to increasing sequencing throughput (Fig. 4). However, other laboratory elements, such as sequencers, robotics/liquid handlers, and computing hardware, were also reported as critical to increasing throughput (Fig. 4).

**Fig. 5:**
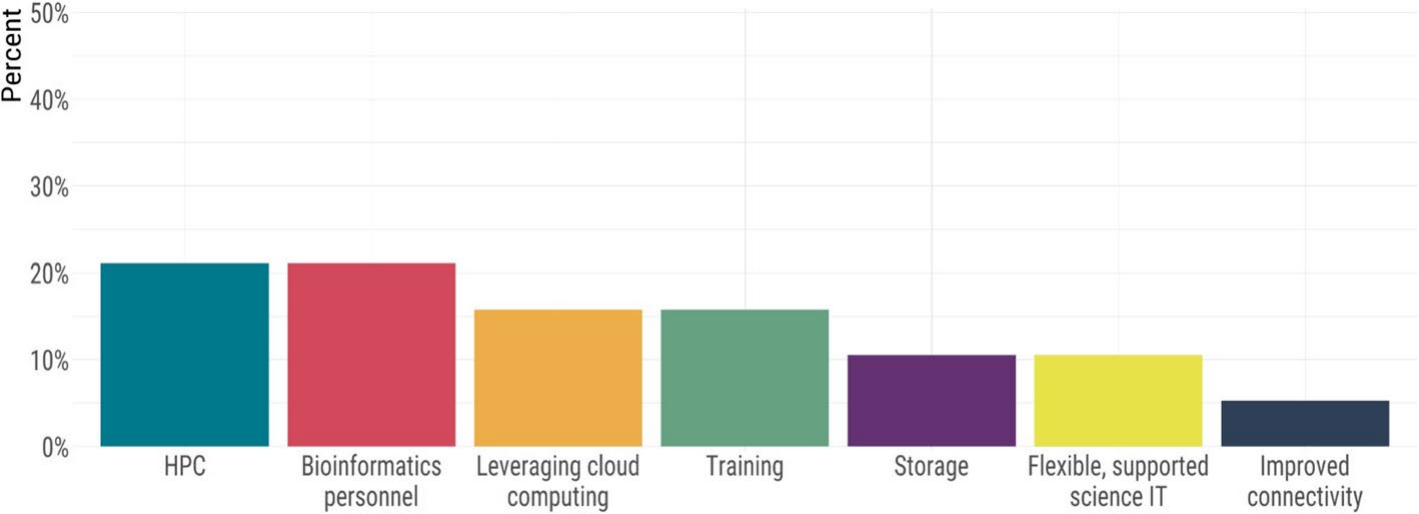
Public health laboratory informatics needs for genomics capacity

**Fig. 6:**
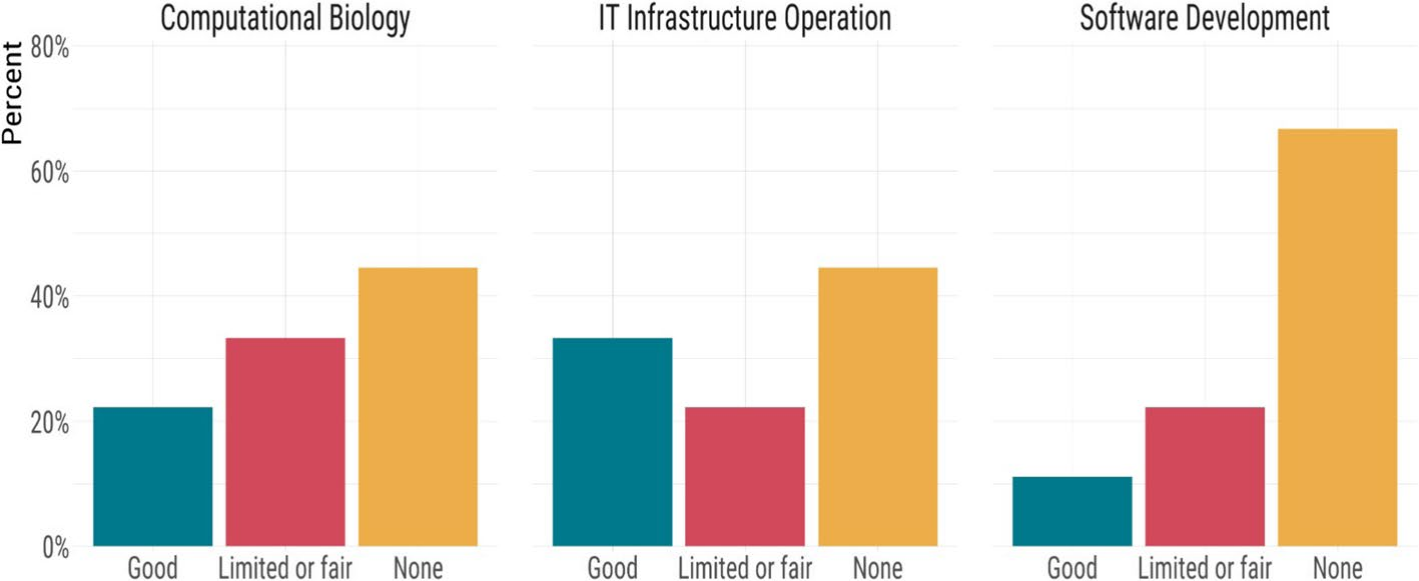
Current availability of informatics expertise in public health laboratories

Probing further into the technical barriers revealed additional pressures in informatics and computational needs. We again identified access to highly qualified bioinformatics personnel as a prominent need (Fig. 5). Other informatics needs identified for the short- and long-term capacity included access to high-performance computing, leveraging cloud computing, improvements to connectivity and storage, and training. Several labs also identified the need for more flexible or nimble scientific IT infrastructure policies and environments (Fig. 5). The participating labs cited the need for expertise in informatics-related disciplines was also identified; the survey revealed that the vast majority of labs had little to no expertise dedicated to scientific software development, computational biology, or the operation of scientific IT infrastructure (Fig. 6). Most computational biologists and bioinformaticians favour the use of Unix-based operating systems; however, our findings indicated that only half of the laboratories utilized Unix-based operating systems (the other half used Windows environments). Furthermore, we found that some laboratories (27%) rely solely on desktop machines for their computing rather than high-performance computing clusters, servers, or cloud computing (see supplemental data). Data management was also described as a significant gap; few reported having a LIMS that interfaced with their genomics infrastructure.

**Fig. 7:**
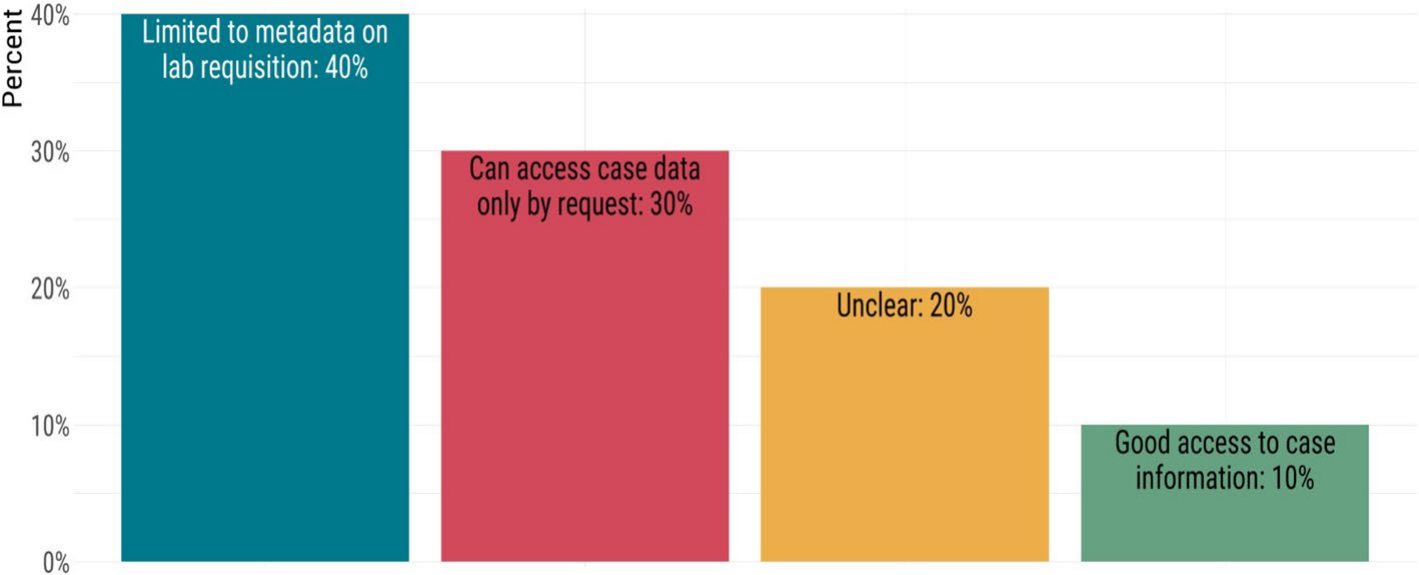
Access to case information (clinical and epidemiological data) by public health laboratories

Routine linking of genomic data with epidemiological or clinical data is highly limited. There was some diversity in the ability of public health laboratories to access case information to make the best interpretation of genomic data. Only 10% of labs reported having good access to epidemiological data, whereas 40% reported that they only have access to information available on a laboratory test requisition form, which is typically highly limited (Fig. 7). Another 30% can access case report information upon request. Many jurisdictions house their laboratory and case data in separate databases, and different public health authorities typically govern each dataset according to jurisdictional privacy legislation. When possible or permitted to do so, accurately linking the genomic and epidemiological data can be challenging and cumbersome to perform. In some instances, laboratories were unsure if a process even exists for them to access epidemiological data (Fig. 7).

**Fig. 8:**
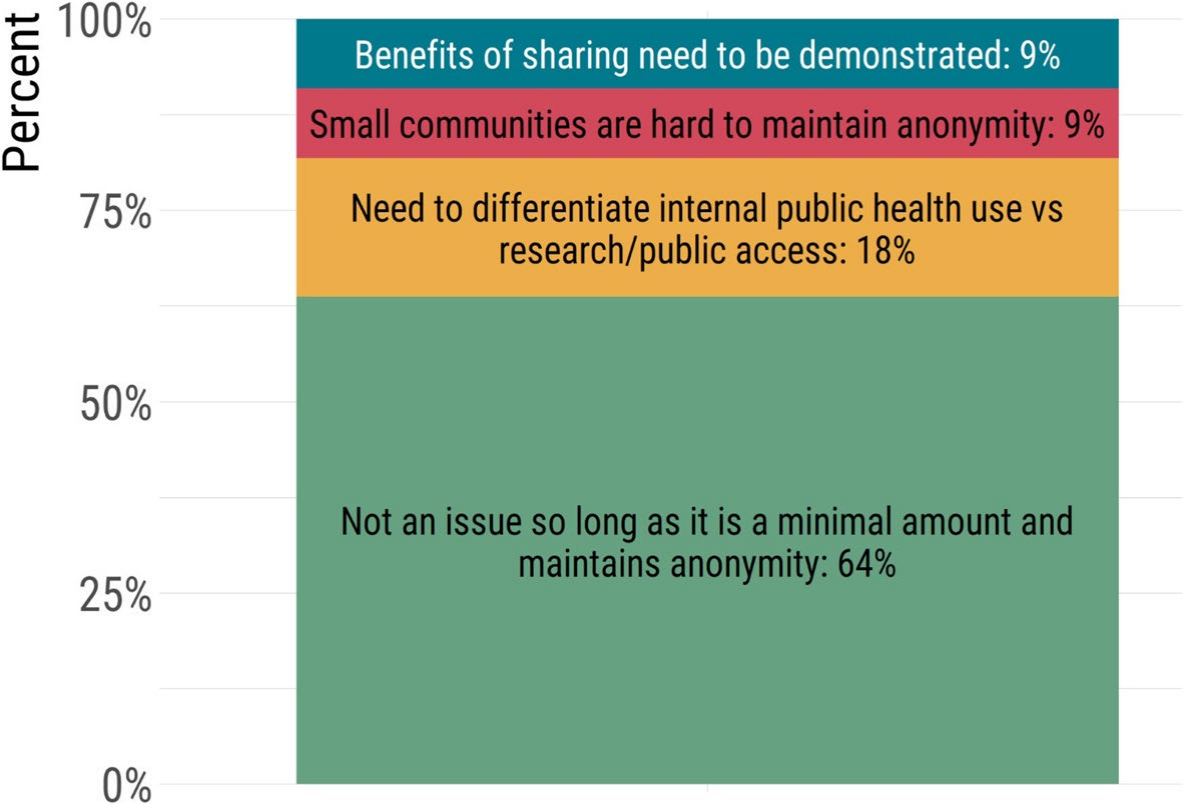
Perceptions of genomic data sharing within public health laboratories

Data sharing is supported, but privacy protections are a major concern. Effective multijurisdictional genomic surveillance requires the rapid sharing of genomic sequence data and accompanying contextual data among jurisdictions while protecting patient privacy. In Canada, large-scale genomic data sharing has little precedent. Before COVID-19, only PulseNet Canada routinely shared surveillance data in a network and submitted genome sequence data to public repositories. Our findings confirm that most public health laboratories support sharing genomic data so long as privacy protections are in place. This typically would manifest as minimal contextual data (e.g., age, sex, collection date, etc.) accompanying the genomic sequence data to maintain patient anonymity (Fig. 8). Another important consideration for sharing genomic data included the need to articulate to decision-makers the benefits of data sharing to the local and global pandemic response. Finally, our findings indicate it is critical to ensure that mechanisms are in place to differentiate data sharing for direct public health use from wider research applications. It is important to note that it can be very difficult to protect privacy when small communities share their data.

The impact of the pandemic on other genomics research and development priorities and operational activities was high. Six months into the pandemic, many (70%) of the public health laboratories had deployed some level of SARS-CoV-2 genomics research and development (Table 1). However, this came at the expense of most other genomics and research activities, with 75% of the laboratories reporting severe impacts on other research areas, with many other projects or activities being suspended entirely. For example, half of the labs relied on personnel previously dedicated to foodborne disease genomic surveillance but had been reassigned to SARS-CoV-2. Public health laboratories sometimes had to rely on PHAC-NML to take over foodborne disease sequencing on their behalf (Table 1).

**Table 1:** Impacts of implementing SARS-CoV-2 genomics on other research, development and surveillance operations

Area of Impact	Proportion of Laboratories
SARS-CoV-2 Genomics R&D activities initiated?	
Yes	70%
No	30%
Other genomics R&D impacted or paused?	
High or severe impact	75%
Minimal or temporary impact	25%
Genomics personnel reassigned from other areas (e.g., foodborne diseases/PulseNet Canada)	
Yes	50%
No	50%

## Discussion

This study of the readiness of Canada’s public health laboratories for performing SARS-CoV-2 genomics identified several key areas to maximize capacity and rapidly build capacity for SARS-CoV-2 sequencing at the massive scale required for pandemic control and response. It is important to note that the scale of the COVID-19 pandemic significantly shifted the perception of what adequate capacity means. While the overall capacity was characterized as low by Canada’s public health laboratories, this study was conducted during the early portion of the pandemic and thus is specifically in the context of a massive pandemic scale. Prior to COVID-19, the scale of capacity building was for numbers of disease cases to be sequenced that were lower by multiple orders of magnitude. Additionally, the attitudes and prioritization of genomic surveillance are likely to have change since this study was conducted. Nonetheless, prioritizing the allocation of resources and new investments according to the needs identified in this study will enable a strategic deployment of solutions in a timely manner for maximum effect at any scale.

A common challenge identified virtually across all laboratories was the lack of bioinformatics expertise and related highly qualified personnel. The ability to recruit and hire highly skilled personnel, or the capacity to train existing personnel, were exposed as major gaps across the country. Many labs have implemented informal means to train personnel in genomics data generation, including competency-based training, development of in-house standard operating procedures, and leveraging knowledge built through training provided by PHAC-NML via PulseNet Canada. However, this is generally more challenging for bioinformatics and data analytics capacity. Specifically, most laboratories cannot hire personnel with bioinformatics or computational biology skills as these skills are rare, and/or laboratories do not have the resources to impart these skills and train personnel from the ground up. Furthermore, certification programs for laboratory technologists in some parts of the country do not include sequencing and/or bioinformatics workflows in their curricula. Few undergraduate or graduate university programs in bioinformatics exist in Canada, further exacerbating the problem into at least the short-term future.

The Canadian public health system operates as a decentralized, federated network of provincial, territorial, local, and federal jurisdictions that work collectively to carry out public health activities to protect the health of Canadians. Public health laboratories engage with their regional health units, private diagnostic laboratories, and PHAC-NML to coordinate and carry out laboratory-based surveillance and response to various infectious diseases, meeting the needs of both local jurisdictions and national public health protection. The federated nature of Canada’s healthcare system places the control and responsibility for delivering public health in the hands of provinces and territories to best meet their specific needs; thus, it has also engendered a fragmented and disjointed federal public health response. Logistical challenges in the timely access of specimens for sequencing can add a considerable lag between sample collection and sequence generation, depending on the centralization/decentralization of the province’s/territory’s infrastructure. In some jurisdictions this lag can be on the order of several weeks. In the absence of these logistical challenges, most, if not all, jurisdictions would likely be capable of generating sequence data with a turnaround time of approximately one to two weeks (from the time of a positive test result), which is the generally accepted turnaround time for effective genomic surveillance [8].

Data sharing also creates significant challenges for effective SARS-CoV-2 genomic surveillance. The contextual data required to perform surveillance resides throughout Canada’s decentralized public health network, which, like specimen collection, can add substantial delays to the collection and transfer of such data to provincial, national, and international databases. In order to properly interpret genomic data, it must be considered in the context of clinical and epidemiological data, i.e., not just the sequence data alone. Linking these data types can be difficult, as they are often collected by different parts of the public health system and often reside separately. Additionally, our study illuminates cultural and organizational barriers to linkage. These issues are not unique to Canada; reports from the United States also indicated that the logistics of getting samples to sequencers as well as the limited access to metadata (spread across the public health system in silos) likely hampered rapid genomics implementation in that country as well [5]. A reluctance to share, based on fears of violating the multitude of privacy policies across jurisdictions, also plays a role. However, it is clear that sequencing alone will not be a valuable complement to the pandemic response; genomics must be linked to clinical and epidemiological data to inform effective public health response activities [33].

High-performance computing and connectivity to enable the analysis and transfer of sequence data and accompanying contextual data to national and international databases will be crucial for the success of Canada’s SARS-CoV-2 genomic surveillance program. Limitations in the local compute capacity add a significant barrier to rapid genomic surveillance; in certain instances, these limitations, including connectivity, were so severe that some labs could not transfer raw sequence data to the national SARS-CoV-2 genomic database housed at PHAC-NML. Most laboratories are supported by an offsite IT team that supports several clients, leading to competing priorities. Most laboratories reported having an enterprise IT infrastructure with restrictive policies that do not accommodate the agile scientific computing environment required to carry out pathogen genomic surveillance. Further, IT policies rarely grant data analysts the technical authority and administrative privileges necessary for professional-grade bioinformatics, which can severely limit the scientific computing capacity available to carry out effective data analysis. Within enterprise IT environments, change management processes and other IT administrative processes are heavy and slow, which is incompatible with the rapid and continuous bioinformatics software development processes required for SARS-CoV-2 genomic data analysis and the analysis of other pathogen genomes. Challenges in IT capital equipment procurement were also reported, with external management and slow response times.

Almost all public health laboratories across Canada had some pre-existing sequencing capacity, largely through participation in previous genomics capacity-building efforts via PulseNet Canada. However, dedicated staff with expertise in foodborne pathogen sequencing in these laboratories have not all been reassigned to the sequencing of SARS-CoV-2 but instead to other pandemic response activities. This left a gap in dedicated personnel to perform SARS-CoV-2 sequencing at a level required to address local and national surveillance priorities. The capacity building provided by PulseNet Canada laid a foundation to build upon but was not designed for the scale needed to support a global pandemic response. Several provinces have access to or have recently acquired new sequencing equipment, which has augmented their capacity since this study was conducted.

When this study was initiated, most public health labs had not implemented routine genomics-based surveillance for SARS-CoV-2, which created a heavy reliance on centralized sequencing at PHAC-NML. Over time, capacity-building efforts to date have resulted in a majority of labs now performing their own sequencing (as of August 2021). Despite this and the other challenges identified by this study, the basic framework to support the successful scale-up of genomic surveillance existed in Canada prior to the start of the pandemic and the Canadian public health system leveraged this capacity. Benchmarks from international partners successful in large-scale genomics implementation are also useful for guiding capacity building in Canada. For example, several of the six key features that enabled the highly successful genomic surveillance implementation in the United Kingdom via the COG-UK Consortium are in place in Canada as well [10]. For example, both Canada and the UK operate as an integrated hub and spoke model; both have implemented a sampling strategy to support representative and targeted surveillance, and both implement a range of technical approaches for sequencing and analysis that build on existing expertise and pipelines. Canada diverges from the COG-UK in the latter’s approach to the logistics of sample and data flow, single-cloud infrastructure, standardized analyses, and integrated genome and patient data [10]. While the national provision of health services in the United Kingdom makes direct comparisons to Canada's public health system difficult, lessons learned from their successes are valuable. The areas where Canada differs from the COG-UK approach are consistent with the common gaps and themes found in this study and further confirm the validity of our findings. Our findings also align with the World Health Organization’s guidance for implementing SARS-CoV-2 genomics for maximum impact [7]. Generally speaking, high-income countries have achieved the highest rate of sequencing, and low-income countries have the least. However, wealth has not been the only predictor of capacity to date, with several low-income countries achieving high sequencing rates, having swiftly adapted expertise from other diseases to SARS-CoV-2 [33]. Public health systems can only fully realize the full benefits of timely SARS-CoV-2 genomic surveillance by ensuring genomics is tied to understanding the biological and clinical impacts and outcomes to direct public health response activities, coupled with rapid and reliable data sharing (with public health authorities and international repositories), and by building expertise, agility and capacity to rapidly detect and determine the significance of new variants [6].

The COVID-19 pandemic has certainly illuminated the pre-existing challenges of building genomics capacity in public health laboratories. Even when the benefits of genomic surveillance are clear and measurable, the public health laboratory typically bears the costs. In contrast, the benefits are largely manifested elsewhere downstream, in the form of illnesses and deaths prevented along with the associated economic and social costs saved. For example, initial PulseNet Canada genomics capacity building largely came from short-term grant money. Despite the significant public health and cost–benefit impact in terms of cases prevented, lives saved, and positive economic results [16, 34], sustainable capacity for personnel and consumables remained elusive prior to COVID-19. While relying on a broader network of academic laboratories and sequencing centers for capacity and expertise during the tremendous response required for the COVID-19 pandemic is appropriate, it must not detract from the critical capacity needed in public health laboratories. During the pandemic, the “all hands on deck” ethos prevailed, uniting all sectors in an effort towards a common goal. However, the core mandates of research and public health institutions differ greatly. When the emergency subsides, public health laboratories must be enabled to provide the protections to which they have been entrusted, and academic institutions’ focus remains on research. The massive redirection of resources within public health laboratories has resulted in the inability to prevent and control other infectious diseases, which puts Canadians at further risk. While Canada has recently invested large amounts of funding to build the landscape of genomics due to the pandemic, the vast majority of it was targeted outside of public health laboratories—to universities, research hospitals, the Canadian Institutes of Health Research, industry, etc. [35]. Fostering a sustainable pathogen genomics program to prevent and control infectious diseases, building from the work done to respond to the pandemic, has health, economic, and broader societal benefit of all Canadians. Funding commensurate with a sustainable genomics program will also ensure that the current capacity does not sit idle once the pandemic subsides. The nature of genomics capacity expertise and infrastructure is such that it cannot be rapidly re-started if it is shut down; the building blocks of capacity must be maintained to be timely and effective when needed.

Canada has taken its first steps towards sustainable pathogen genomic surveillance through the newly enacted CPHLN Covid Genomics Program (CCGP), which aims to build capacity and resilience directly in the public health laboratories by addressing the need for highly qualified personnel in genomics and data analytics. A key part of this program is the placement of Genomics Liaison Technical Officers from the National Microbiology Laboratory in provincial public health laboratories. Embedding these highly qualified federal personnel provides the technical expertise needed in public health laboratories and enables the coordination of activities across laboratories for a cohesive and effective national response. This strengthened capacity would allow the use of genomics to protect Canadians from all infectious disease threats. Sustainable genomics capacity is well-aligned with the new Pan-Canadian Health Data Strategy, and this study’s results are consistent with many of the root causes identified in the Expert Advisory Group’s first report and will contribute to the vision of achieving a fully integrated and continuously optimized health data ecosystem [36].

## Conclusions

Despite the challenges and barriers, Canada was ultimately able to launch genomic surveillance for SARS-CoV-2 via the CanCOGeN program. The findings of this study guided capacity building efforts, which included activities such as centralization of sequencing at PHAC-NML and academic partners during the initial phases, the centralization of analysis at PHAC-NML and through CanCOGeN, the deployment of highly qualified personnel via the CCGP program, and the illumination of logistical challenges for dedicated focus. In the post-pandemic era, re-assessing the capacity for pathogen genomic surveillance in Canada’s public health laboratories will be useful to measure the overall impact and increase in capacity.

The results of this study will be valuable to guide the creation of sustainable national pathogen genomic surveillance that can be applied to control the spread of other infectious diseases circulating in Canada or that may emerge in the future. This study finds that the sequencing itself is not the limiting factor per se; all of the steps preceding the sequencing (specimen logistics, etc.) and the post-sequencing work (computational biology, etc.) are the major bottlenecks that must be addressed. Moreover, communication channels and relationships are just as critical as the science and technology elements for data sharing and linkage of laboratory and epidemiological data, now more than ever. While SARS-CoV-2 surveillance has dominated the discussion on genomics capacity building in Canada, it is important to highlight that genomics capacity will foster rapid and effective response for all infectious diseases. This is particularly critical as we prepare for new infectious disease challenges that will emerge in the era of climate change and its impact on zoonotic infections. Finally, the unique qualitative study design enabled the nuances of genomics implementation to be captured in-depth in a way that quantitative surveys may not. This study and its methodology may be useful for other countries and laboratory networks struggling to prioritize capacity-building activities for pathogen genomics.

## Supplementary Information

**Additional file 1.** Full data set: themes and responses from focus group discussions.

## Data Availability

All data generated or analysed during this study are included in this published article [and its supplementary information files].

## Abbreviations

SARS-CoV-2: Severe acute respiratory syndrome coronavirus 2
COVID-19: Coronavirus disease pandemic that began in 2019
IRIDA: Integrated Rapid Infectious Disease Analysis platform for genomic epidemiology
PHAC-NML: Public Health Agency of Canada’s National Microbiology Laboratory
CanCOGeN: Canadian COVID Genomics Network
CPHLN: Canadian Public Health Laboratory Network
IT: Information Technology
LIMS: Laboratory Information Management Systems
R&D: Research and Development
COG-UK: COVID-19 Genomics United Kingdom Consortium
